# Supplementary material for: Characteristic of Metabolic Status in Heart Failure and Its Impact in Outcome Perspective
Source: Metabolites. 2020 Oct 29;10(11):437. doi: 10.3390/metabo10110437 (PMC7692076; doi:10.3390/metabo10110437)
Supplement: Supplementary file 1 [file metabolites-10-00437-s001.pdf]

Supplementary Data

Supplementary Table S1. Receiver operating characteristics (ROC) curves with area under curve (AUC) of single or combined metabolites to discriminate event and non-event patients in heart failure.

Metabolites	AUC	95% CI
3-Methylglutarylcarnitine	0.763	0.64-0.88
Decatrienoylcarnitine	0.733	0.60-0.86
Tetradecenoylcarnitine	0.728	0.60-0.86
Dimethyluric acid	0.722	0.60-0.85
Dimethylxanthine	0.720	0.59-0.85
LysoPC(18:2)	0.706	0.57-0.84
LysoPE(18:2)	0.700	0.56-0.84
Phenylacetylglutamine	0.699	0.56-0.84
Dodecenoylcarnitine	0.685	0.55-0.82
3-Hydroxyoctanoylcarnitine	0.678	0.54-0.82
LysoPC(16:0)	0.678	0.54-0.82
LysoPC(14:0)	0.678	0.54-0.82
Tryptophan	0.673	0.54-0.8
LysoPS(18:1)	0.671	0.53-0.82
Tetradecadiencarnitine	0.666	0.53-0.80
LysoPC(15:0)	0.661	0.52-0.80
LysoPC(20:5)	0.625	0.48-0.77
Butyrylcarnitine	0.617	0.47-0.77
Hypoxanthine	0.604	0.46-0.75
BNP	0.602	0.45-0.75
**Combinations**		
Tetradecenoylcarnitine+Dimethylxanthine+Phenylacetylglutamine+Hypoxanthine+BNP	0.874	0.78-0.97
Tetradecenoylcarnitine+Dimethylxanthine+ Phenylacetylglutamine+Hypoxanthine	0.871	0.78-0.96
Tetradecenoylcarnitine+ Dimethylxanthine+ Hypoxanthine	0.860	0.77-0.95
Tetradecenoylcarnitine+Phenylacetylglutamine+Hypoxanthine	0.856	0.76-0.95
Decatrienoylcarnitine+Dimethylxanthine+Hypoxanthine	0.850	0.76-0.94
Tetradecenoylcarnitine+Dimethyluric acid+ Hypoxanthine	0.844	0.75-0.94
Tetradecenoylcarnitine+ Dimethylxanthine+ Phenylacetylglutamine	0.838	0.73-0.94
Tetradecenoylcarnitine+Dimethyluric acid+ Phenylacetylglutamine	0.830	0.73-0.94
Decatrienoylcarnitine+Dimethyluric acid+Hypoxanthine	0.842	0.75-0.94
3-Methylglutarylcarnitine + Dimethyluric acid+ Dimethylxanthine	0.816	0.71-0.92
Decatrienoylcarnitine+Phenylacetylglutamine+Hypoxanthine	0.812	0.71-0.92
Decatrienoylcarnitine+Dimethyluric acid+Phenylacetylglutamine	0.798	0.69-0.91
Decatrienoylcarnitine+Dimethylxanthine+Phenylacetylglutamine	0.795	0.68-0.91
3-Methylglutarylcarnitine + Dimethylxanthine	0.815	0.71-0.92
Decatrienoylcarnitine+Hypoxanthine	0.813	0.71-0.92
3-Methylglutarylcarnitine + Dimethyluric acid	0.808	0.70-0.91
Tetradecenoylcarnitine+ Dimethylxanthine	0.804	0.69-0.92

Tetradecenoylcarnitine+ Phenylacetylglutamine	0.791	0.68-0.91
Tetradecenoylcarnitine+Dimethyluric acid	0.790	0.68-0.90
Tetradecenoylcarnitine+ Hypoxanthine	0.798	0.68-0.91
Decatrienoylcarnitine+Dimethylxanthine	0.797	0.69-0.91
Decatrienoylcarnitine+Dimethyluric acid	0.786	0.67-0.90
Decatrienoylcarnitine+Phenylacetylglutamine	0.751	0.63-0.87

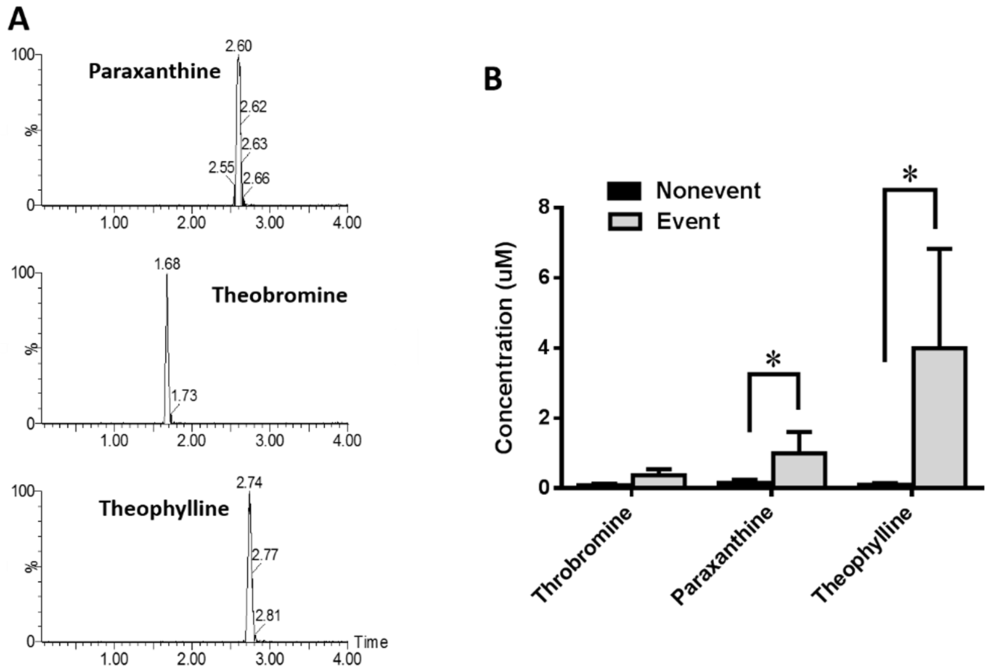

Supplementary figure S1. Levels of dimethylxanthines in event and ono-event groups. (A) Chromatography of paraxanthine, therobromine, and theophylline. (B) Concentration of therobromine, paraxanthine, and theophylline in event (n=20) and non-event (n=22) groups. Data were compared by Mann-Whitney U. *p < 0.05
